# Impact of COVID-19 pandemic waves on colorectal cancer stage migration and diagnostic delays

**DOI:** 10.3389/fonc.2026.1837204

**Published:** 2026-07-29

**Authors:** Klaudia Széphelyi, Adrienn Biró, József Tollár

**Affiliations:** 1 Doctoral School of Health Sciences, Faculty of Health Sciences, University of Pécs, Pécs, Hungary; 2 Somogy County Kaposi Mór Teaching Hospital, Kaposvár, Hungary; 3 Digital Development Center, Széchenyi István University, Győr, Hungary; 4 Faculty of Health Sciences, University of Pécs, Pécs, Hungary

**Keywords:** cancer staging, colorectal cancer, COVID-19, diagnostic delay, follow-up, magnetic resonance imaging, pandemic waves

## Abstract

**Introduction:**

The COVID-19 pandemic was associated with substantial changes in cancer diagnosis and treatment. This study aimed to assess changes in colorectal cancer diagnostics during the COVID-19 pandemic by analyzing changes in tumor stage and the number of newly diagnosed cases across different pandemic waves and the post-COVID period using MRI-based data.

**Methods:**

The study included 198 patients with colorectal cancer, 43 with a new diagnosis. The study period was divided into five COVID-19–related intervals. The number of newly diagnosed cases across COVID-19 waves was compared using one-way ANOVA. Stage distribution differences were assessed using Fisher’s exact test. In follow-up patients, changes were analyzed using the Wilcoxon signed-rank test. Secondary outcomes included tumor changes, number of missed treatments, and the presence of metastases.

**Results:**

Monthly averages of newly diagnosed colorectal cancer cases were lower during the COVID-19 waves than in the post-COVID period (p < 0.001). Although overall tumor stage did not differ between waves, larger tumors were more frequent during the COVID-19 waves, whereas early-stage (T1) tumors were more common in the late/post-COVID period (p < 0.001). Tumor stage distribution also differed between the COVID and post-COVID periods, with a shift toward earlier-stage disease after the pandemic (p = 0.002). No differences were observed in missed treatments across COVID-19 periods (p-value > 0.05).

**Discussion:**

The COVID-19 pandemic was associated with a lower number of newly diagnosed colorectal cancer cases during the pandemic and a higher proportion of patients presenting with more advanced disease stages. These findings suggest an association with pandemic-related healthcare disruption; however, causality cannot be established.

**Trial registration:**

https://clinicaltrials.gov/, identifier NCT07328399.

## Graphical abstract

**Figure:**
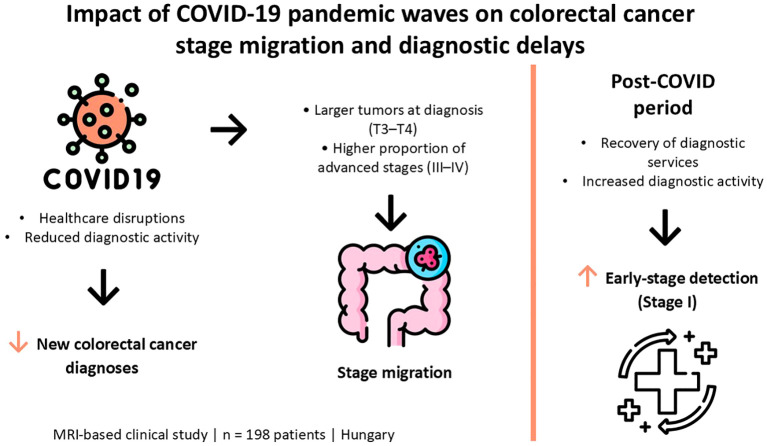

## Highlights

The COVID-19 pandemic placed considerable pressure on healthcare systems, raising concerns about delays in cancer diagnosis and treatment. Early detection is critical in colorectal cancer, as diagnostic and treatment delays are associated with a poorer prognosis. In this study, we examined the association between different waves of the COVID-19 pandemic and diagnostic delays and tumor stage at presentation in patients with colorectal cancer. By analyzing data from newly diagnosed patients and those under follow-up before and during the pandemic, we identified significant changes in the timing of diagnosis and in the distribution of disease stages. Our findings suggest that healthcare disruptions during public health crises may be associated with changes in colorectal cancer diagnostic activity and underline the importance of maintaining timely cancer diagnostic services even during emergencies.

## 1 Introduction

COVID-19 (Coronavirus Disease 2019), caused by the SARS-CoV-2 virus, posed a serious challenge for global healthcare systems (1). Symptoms included fever, cough, general weakness or fatigue, changes or loss of taste and smell, sore throat, headache, muscle aches, and diarrhea. The most vulnerable groups were people over 60 years of age, pregnant women, and individuals with underlying medical conditions, such as obesity, diabetes, or heart disease. Most people infected with COVID-19 were asymptomatic or had mild to moderate respiratory illness and recovered without specific treatment (2).

The COVID-19 pandemic has affected various areas of healthcare, including cancer diagnosis and treatment. The overburdened healthcare system, avoidance of hospital visits due to the risk of infection, and the implementation of epidemiological restrictions led to significant changes in patient care and cancer detection patterns. During the pandemic, limited access to diagnostic procedures, the suspension of screening programs, and the overall overload of healthcare services substantially affected patient care. As a consequence, many patients experienced delays in diagnosis and treatment, which may have had long-term implications for disease outcomes (3, 4).

The incidence of colorectal cancer in Hungary exceeds the EU (European Union) average, and colorectal cancer–related mortality rates are also among the highest in the country (5). According to the KSH (Central Statistical Office), the leading causes of death in Hungary in 2024, in addition to cardiovascular diseases, were cancer (6). Based on data from the National Cancer Registry, in 2022, lung, prostate, and colorectal cancers were the most common among men, and breast, lung, and colorectal cancers were the most common among women (7).

Due to the measures taken to combat the COVID-19 pandemic, not only newly suspected cancer patients but also those already diagnosed and undergoing treatment faced difficulties. In addition to the periodic suspension of screening and preventive activities and delays in the initiation of therapy, the continuity of care was also disrupted. These interruptions were only partially mitigated by the reorganization of healthcare services, including the increased use of telemedicine (3).

Although several studies have examined the impact of the COVID-19 pandemic on colorectal cancer diagnosis, most have relied on registry-based data and compared only pre-pandemic and pandemic periods. In contrast, the present study offers a detailed MRI (Magnetic Resonance Imaging)-based analysis across individual COVID-19 waves and the post-COVID period, distinguishing between newly diagnosed and follow-up patients and providing insight into diagnostic delays, stage migration, and treatment disruptions within real-world clinical practice. To our knowledge, this is the first study from Hungary to provide an MRI-based, wave-specific analysis of the impact of the COVID-19 pandemic on colorectal cancer diagnostics, including the post-COVID period.

## 2 Materials and methods

### 2.1 Participants and design

Patients were recruited from the outpatient department of Szent Margit Hospital (Budapest, Hungary) between September 2020 and December 2023.

The target population comprised patients with colorectal cancer examined in the MRI unit of Szent Margit Hospital. The inclusion criterion was an MRI referral indicating suspected colorectal malignancy, with the referring physician sending the patient for examination due to a suspected tumor. Exclusion criteria were cases in which Szent Margit Hospital did not issue the referral. A total of 198 participants were enrolled in the study, of whom 43 participants were newly diagnosed. The mean age of the participants was 65.84 ± 12.22 years (age range 33–90 years).

We reviewed the MRI findings of colorectal cancer patients and collected demographic data (age and gender), as well as information on newly diagnosed colorectal cancers. In the follow-up group, we also examined previous imaging studies (CT – Computer Tomography and MRI), recorded the dates of the examinations, and documented the tumor stage and TNM (Tumor-Node-Metastasis) classification at that time. Based on the current MRI and the most recent CT studies available in the medical history, we determined the latest TNM stage. CT scan is used as the imaging modality of choice for the staging and follow-up of colon cancer, while the staging of rectal cancer normally involves MRI scan due to higher accuracy of local staging. As all participants of this study had undergone MRI scanning at our center, MRI was the sole imaging modality that could be used consistently in all patients. Thus, radiological evaluation based on MRI results was chosen as the radiological gold standard for ensuring the methodology consistency in the whole study. Whenever possible, prior CT scans were evaluated together with MRI findings to determine TNM stage and assess the progress of the disease during the follow-up period.

We compared the results of previous imaging studies and documented whether the tumor had changed, and if so, whether it had progressed or regressed. We also recorded the time interval (in months) between the two examinations. By reviewing oncology outpatient records provided in the patient history, we assessed the difficulties caused by the epidemic in tumor management, including whether any treatments had been missed. In the follow-up groups, we also monitored whether the MRI-diagnosed tumor had relapsed.

The study period was divided into five predefined time periods based on official epidemiological data published by the NNGYK (National Public Health Center of Hungary) and the WHO (World Health Organization) (8, 9). These periods included four COVID-19 pandemic waves and one post-COVID period:

- September 2020 – January 2021 (4 months) COVID-19 Wave 2,
- February 2021 – September 2021 (8 months) COVID-19 Wave 3,
- September 2021 – January 2022 (4 months) COVID-19 Wave 4,
- January 2022 – April 2022 (3 months) COVID-19 Wave 5,
- April 2022 – December 2023 (21 months) post-COVID period.

We were unable to examine Wave 1 of the COVID-19 pandemic because the MRI unit at Szent Margit Hospital only began operating during the pandemic, in September 2020.

### 2.2 MRI protocol

All MRI examinations were performed using a 1.5-T GE Signa Voyager scanner (GE HealthCare, Milwaukee, WI, USA). For co using gadoteric acid (Dotarem, Guerbet, France) when clinically indicated according to routine institutional practice. The routine pelvic MRI protocol included the following imaging sequences:

- Sagittal T2-weighted imaging;
- Coronal T2-weighted imaging;
- Axial T2-weighted imaging;
- Axial T2-weighted fat-saturated imaging;
- Axial T1-weighted imaging;
- Axial T1-weighted fat-saturated imaging;
- Coronal T2-weighted fat-saturated imaging;
- Axial diffusion-weighted imaging (DWI);
- Post-contrast 3D axial T1-weighted imaging;
- Post-contrast sagittal T1-weighted fat-saturated imaging;
- Post-contrast coronal T1-weighted fat-saturated imaging;
- Post-contrast axial T1-weighted imaging.

### 2.3 Outcomes

The primary outcomes were the TNM classification and overall stage of colorectal cancer during the four waves of the COVID-19 pandemic, with respect to the number of new diagnoses and follow-up patients in each of the four waves and the post-pandemic period.

Tumor stage was determined according to the AJCC.(American Joint Committee on Cancer) 8th edition TNM classification using the MRI examination together with available previous CT examinations and clinical records (**Supplementary Table 1**) (10). As this was a retrospective real-world study, MRI examinations were interpreted as part of routine clinical practice by board-certified radiologists employed by the reporting service contracted by the institution. The examinations were not re-evaluated specifically for this study. Instead, the original clinical radiology reports and imaging findings were retrospectively reviewed and extracted from the medical records.

Secondary outcomes included tumor progression or regression, number of missed treatments, and the presence and distribution of metastases.

Tumor progression was defined as an increase in TNM stage compared with the previous available examination, taking all TNM components into account. Regression was defined as a decrease in TNM stage, while stable disease was defined as no change in TNM stage between consecutive examinations. Tumor recurrence was recorded when recurrent colorectal cancer was explicitly documented in the radiology report. Missed treatment (e.g., surgery, chemotherapy, radiotherapy, follow-up imaging, or outpatient oncologic visits) was defined as documented interruption, postponement, or non-participation in planned oncological treatment due to the COVID-19 pandemic, as recorded in the patients’ medical history or outpatient records. Outcome classification was performed retrospectively by the investigators based on the original radiology reports and corresponding outpatient medical documentation.

### 2.4 Statistical analysis

Baseline characteristics were summarized using descriptive statistics. Continuous variables are presented as mean ± SD (standard deviation), while categorical variables are presented as frequency and percentage [n (%)].

Baseline demographic variables were compared across predefined time periods (COVID-19 pandemic waves and the post-COVID period) to test the distribution of age and sex between the groups. Age differences were evaluated using an independent-samples t-test, while sex distribution was examined using the chi-square test. Normality testing was performed only on the baseline descriptive variable (age) to inform the choice of parametric or nonparametric tests. As the main outcomes of the study were categorical or ordinal in nature, normality assumptions were not relevant for the primary analyses.

The main analytical design was based on a time factor consisting of five time periods, corresponding to the four COVID-19 pandemic waves and the post-COVID period. Wave merging was performed consistently across subgroup analyses when required due to low cell counts.

To evaluate changes in the number of newly diagnosed colorectal cancer cases across the COVID-19 waves and the post-COVID period, a one-way ANOVA (analysis of variance) was performed. Given the relatively small number of newly diagnosed cases, this analysis should be considered exploratory and hypothesis-generating. Differences in stage distribution of newly diagnosed tumors across the COVID waves, as well as the proportion of missed oncologic treatments, were analyzed using Fisher’s exact test.

For follow-up patients, the tumor stage determined at the most recent prior imaging examination was compared with the stage obtained at the current MRI examination using the Wilcoxon signed-rank test, reflecting paired ordinal data. Differences in tumor size category distributions between the COVID and post-COVID periods were examined using the chi-square test.

For statistical analysis, we grouped the second and third waves as the first COVID waves, and the fourth and fifth waves as the late COVID and post-COVID periods. In addition, because the number of patients within each stage subgroup was relatively small, stages were combined for analysis.

Due to the limited number of cases in several subgroups, COVID-19 pandemic waves were merged into early (second–third) and late/post-COVID (fourth–fifth and post-COVID) periods, and tumor stage categories were collapsed for inferential statistical analyses. These decisions were made to ensure adequate cell counts and reduce sparse data bias, while unaggregated data are presented in the tables to preserve transparency. Inferential statistical tests were applied only when the assumptions of the selected tests were met; otherwise, analyses were restricted to descriptive reporting. These groupings were applied solely for statistical purposes and should not be interpreted as implying epidemiological equivalence, as the individual pandemic waves differed in their epidemiological characteristics and predominant SARS-CoV-2 variants.

No formal correction for multiple comparisons was applied for the primary analyses, as these were exploratory and hypothesis-generating in nature. However, for the one-way ANOVA comparing monthly averages of newly diagnosed cases across time periods, a *post hoc* Bonferroni correction was applied to account for multiple pairwise comparisons.

A two-sided significance threshold of p-value was applied for all statistical tests; p-values are reported as exact values or thresholds (e.g., p-value < 0.001) where appropriate. Statistical analyses were performed using IBM SPSS Statistics for Windows, Version 26.0 (IBM Corp., Armonk, NY, USA).

### 2.5 Ethical considerations

This study was retrospectively registered in ClinicalTrials.gov (identifier: NCT07328399) on January 3, 2026. The registration was performed after completion of data collection because this was a retrospective observational study based on existing clinical records. Ethical approval was obtained prior to data collection from the Regional Research Ethics Committee of Szent Margit Hospital (IG/2037-1/2023). The study was conducted according to the World Medical Association Declaration of Helsinki. Due to the retrospective study design, informed consent was waived in accordance with institutional guidelines. Data handling and collection procedures complied with General Data Protection Regulation (GDPR) and patient confidentiality regulations.

## 3 Results

### 3.1 Baseline characteristics

Baseline characteristics of the entire study population are summarized with detailed descriptive data stratified by diagnostic status and sex provided in **Supplementary Table 2**. The cohort involved 90 male and 108 female participants (age: 65.84 ± 12.22, range: 33 to 90 years). The study cohort comprised 198 patients, including 155 follow-up patients and 43 newly diagnosed colorectal cancer patients.

### 3.2 Primary outcomes

#### 3.2.1 Overall study population

To assess the extent to which the number of newly diagnosed colorectal tumors decreased during the COVID-19 pandemic, we examined the number of new cases identified in each COVID wave and in the post-COVID period. **Table 1** presents the distribution of newly diagnosed cases across the different waves. Percentages of newly diagnosed cases are based on the total number of newly diagnosed patients (n = 43).

**Table 1:** The distribution of newly diagnosed cases across the different waves (n=43).

Period of examination		Total cases, n	Newly diagnosed cases, n (%)	Average new cases per month
During COVID				
Second wave		29	5 (11.6%)	1.25
Third wave		27	5 (11.6%)	0.63
Late-COVID period		38	8 (18.6%)	1.14
	Fourth wave	26	7 (16.3%)	1.75
	Fifth wave	12	1 (2.3%)	0.33
Post-COVID period		104	25 (58.1%)	1.19

**Figure 1:**
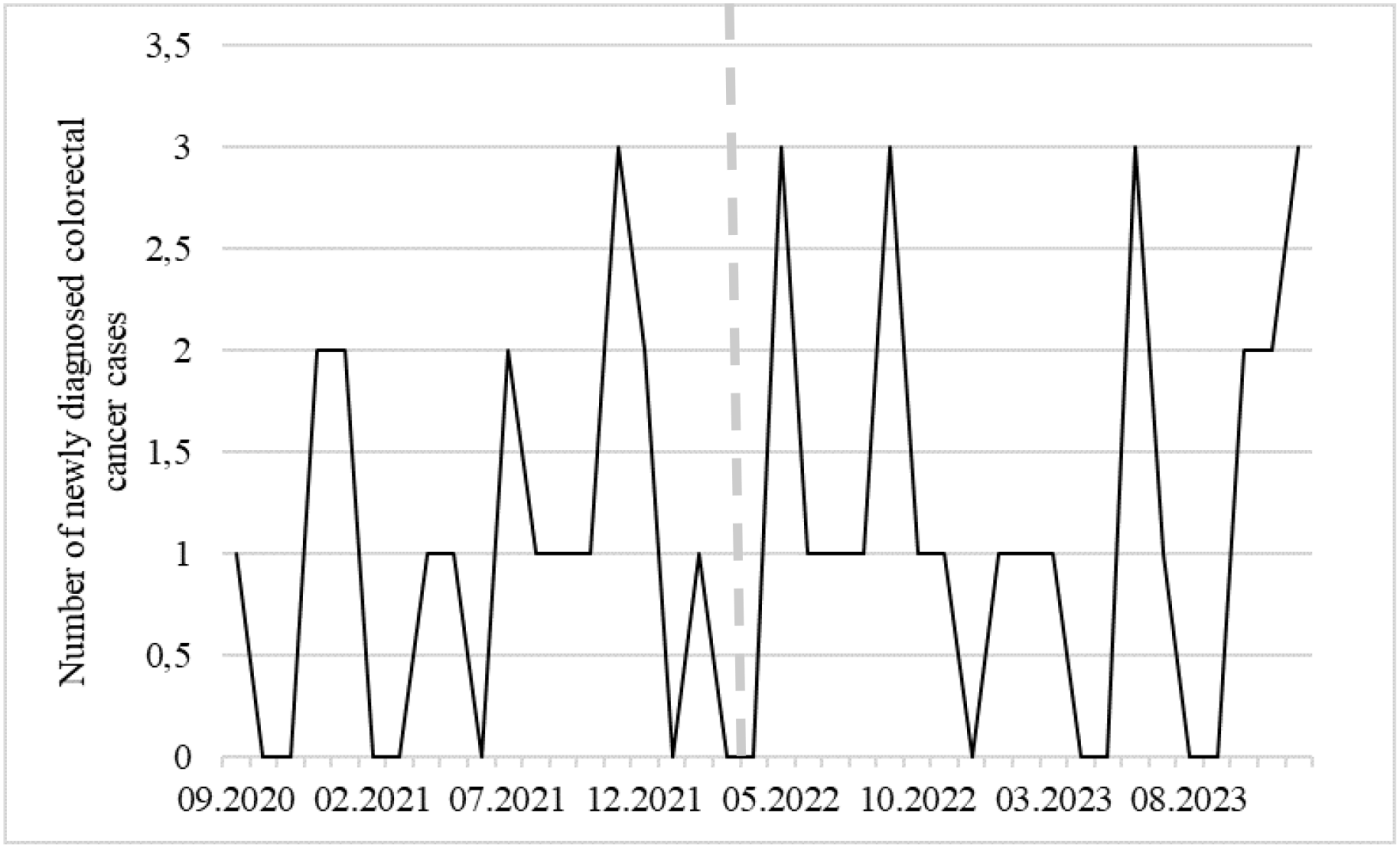
Number of new cases during the COVID-19 waves and the post-COVID period. The vertical line indicates the beginning of the post-COVID period (April 2022). Zero values indicate months in which no newly diagnosed cases were recorded.

Since the lengths of the periods differed, monthly averages were calculated to obtain more accurate results. Due to the low number of cases in the fifth COVID-19 wave (n = 1), the fourth and fifth waves were merged and analyzed as a late COVID period for statistical analysis. A one-way ANOVA demonstrated significant differences in the monthly averages of newly diagnosed colorectal cancer cases across COVID-19 waves (p<0.001). *Post hoc* analysis with Bonferroni correction showed that the third COVID-19 wave was associated with a significantly lower monthly number of newly diagnosed cases compared with the second wave, the late COVID period, and the post-COVID period (all p-value < 0.001). No significant differences were observed between the late COVID and post-COVID periods. (**Figure 1**).

**Table 2** shows the aggregated stage distribution of newly diagnosed colorectal cancer cases across early and late/post-COVID periods, corresponding to the groups used in Fisher’s exact test. Percentages are based on the total number of newly diagnosed patients (n = 43). Detailed wave-specific data are provided in **Supplementary Table 3**.

**Table 2:** The stage distribution of newly diagnosed colorectal cancer cases across the different COVID-19 waves (n=43).

Stage	COVID first waves, n (%)	COVID late waves + post-COVID period, n (%)	Total, n (%)
Stage I.	3 (7%)	16 (37.2%)	19 (44.2%)
Stage II.	2 (4.7%)	3 (7%)	5 (11.6%)
II./A	2 (4.7%)	2 (4.7%)	4 (9.3%)
II./B	0	0	0
II./C	0	1 (2.3%)	1 (2.3%)
Stage III.	5 (11.6%)	14 (32.6%)	19 (44.2%)
III./A	1 (2.3%)	7 (16.3%)	8 (18.6%)
III./B	2 (4.7%)	4 (9.3%)	6 (14%)
III./C	2 (4.7%)	3 (7%)	5 (11.6%)
Stage IV.	0	0	0

Based on the unaggregated data presented in **Table 2**, COVID-19 waves were grouped into early (second–third) and late/post-COVID (fourth–fifth and post-COVID) periods, and tumor stages were collapsed into four categories (I–IV) for statistical analysis due to the low number of cases in several subgroups. Differences between groups were analyzed using Fisher’s exact test, which did not reveal any statistically significant results (p> 0.05).

We examined tumor size in both newly diagnosed cases and in patients with previously known tumors, with the aggregated distributions shown in **Table 3** (detailed wave-specific data are provided in **Supplementary Table 4**). Percentages are based on the total study population (n = 198).

**Table 3:** Tumor size distribution during early and late/post-COVID periods in the whole sample (n = 198).

Tumor size	COVID first waves, n		COVID late waves + post-COVID period, n			Total
	Wave 2	Wave 3	Wave 4	Wave 5	Post-COVID	
T1	22 (11.1%)		102 (51.5%)			124 (62.6%)
T2	8 (4%)		16 (8%)			24 (12.1%)
T3	13 (6.6%)		15 (7.5%)			28 (14.4%)
T4	13 (6.6%)		9 (4.5%)			22 (11.1%)
T4a	10 (5%)		6 (3%)			16 (8%)
T4b	3 (1.5%)		3 (1.5%)			6 (3%)

**Table 3** presents tumor size distributions across individual COVID-19 waves and the post-COVID period; for statistical analysis, COVID-19 waves were grouped into early and late/post-COVID periods as described below. Because the number of patients in each subgroup was relatively small, both tumor size categories (T1, T2, T3, T4) and COVID-19 waves (first and late/post) were combined for analysis. Confirmed by the chi-square test, there was a statistically significant difference in the distribution of tumor size categories between the COVID first waves and late/post-COVID periods (p-value < 0.001). During the COVID period, a higher proportion of patients presented with larger tumors, whereas smaller tumors were more frequently observed in the post-COVID period. No *post-hoc* analysis was performed, as comparisons were limited to these two aggregated time periods. Examining both follow-up and newly diagnosed patients together, **Table 4** shows—and the chi-square test confirms—that there was a statistically significant difference in the distribution of tumor stages between the COVID and post-COVID periods (p-value < 0.001). Percentages are based on the total study population (n = 198). During the COVID period, a higher proportion of patients were diagnosed with advanced-stage disease, whereas in the post-COVID period, a substantially larger number of patients were diagnosed at an early stage.

**Table 4:** The distribution of tumor stages between the COVID and post-COVID periods in the whole sample (n=198).

Tumor stage	COVID first waves, n (%)	COVID late waves + post-COVID period, n (%)
Stage I.	33 (16.6%)	75 (37.9%)
Stage II.	12 (6.1%)	5 (2.5%)
Stage III.	16 (8.1%)	13 (6.5%)
Stage IV.	33 (16.6%)	11 (5.5%)

### 3.3 Secondary outcomes

Of the 155 patients who attended follow-up examinations, tumor stage worsened in 31 cases, improved in 66 cases, and remained unchanged in 58 cases. For this analysis, we compared the tumor stage recorded at the most recent previous imaging examination with the stage determined after the current MRI using the Wilcoxon Signed-Rank test. The statistical test showed a significant difference between the previous and current stage classifications, indicating measurable progression or regression (p-value = 0.002). The average time between the two examinations was 8.95 months (± 8).

Overall, 9 of the 155 follow-up patients (5.8%) experienced at least one missed treatment. During the COVID-19 period, 6 of 76 follow-up patients (7.9%) missed treatment, compared with 3 of 79 patients (3.8%) during the post-COVID period (**Table 5**). Percentages are calculated within each study period. Fisher’s exact test showed no statistically significant difference between the study periods (p > 0.05).

**Table 5:** Missed treatments in the follow-up group (n=155).

COVID period	No missed treatment (cases), n	Missed treatment (cases), n	% missed treatment
During COVID	70	6	7.9%
Second wave	21	3	12.5%
Third wave	20	2	9.1%
Fourth wave	18	1	5.3%
Fifth wave	11	0	0
Post-COVID period	76	3	3.8%

## 4 Discussion

According to our hypothesis, the COVID-19 pandemic was associated with changes in the diagnosis, monitoring, and progression of colorectal cancer. Most studies focus primarily on changes in screening volume, whereas our analysis aimed to examine colorectal cancer more comprehensively — including newly diagnosed and follow-up cases, tumor stage, TNM classification, and the clinical impact of missed treatments.

In the whole sample, 43 patients were newly diagnosed with colorectal cancer. During the COVID waves, the average monthly number of cases was approximately 20% lower compared with the post-COVID period. These findings suggest a relative increase in diagnostic activity in the post-COVID period compared with the COVID waves, however, this observation should be interpreted cautiously due to the limited number of newly diagnosed cases. Our findings are consistent with previous reports showing declines in colorectal cancer diagnoses during the pandemic. In another study, similar to ours, the number of screening tests – and consequently the number of newly diagnosed colorectal cancer cases – decreased significantly during the COVID period, with reported reductions typically ranging between 20% and 40%, resulting in a higher proportion of cancers being diagnosed at more advanced stages (11–14). Recent studies likewise reported a substantial decline in colorectal cancer diagnostic activity during the pandemic, accompanied by an increase in advanced-stage and larger tumors, and a subsequent rebound in early-stage diagnoses in the post-COVID period (15–18). Our data are in line with the Dutch findings: we also saw a reduction in new diagnoses during the COVID waves and a subsequent increase post-pandemic (19). Across these studies, reported reductions in newly diagnosed colorectal cancer cases during the pandemic typically ranged between 20% and 40%, with a parallel shift toward higher-stage disease at diagnosis.

Regarding the stage distribution of newly diagnosed colorectal cancer cases, no statistically significant differences were observed across the COVID-19 waves, as indicated by Fisher’s exact test. Nevertheless, descriptively, advanced-stage tumors (stage III) accounted for 33–40% of cases during the COVID waves, compared with approximately 29% in the post-COVID period. Although we did not find a significant association between tumor stage and the COVID waves, other studies have demonstrated that more advanced-stage tumors were diagnosed during the first year of the pandemic (12). An apparent discrepancy was observed between the newly diagnosed and follow-up cohorts. Although no Stage IV disease was identified among newly diagnosed patients, the proportion of Stage IV disease increased within the follow-up cohort over time. One possible explanation is that pandemic-related disruptions may have affected patients who were already undergoing oncologic follow-up more than those presenting with an initial diagnosis. Delays in routine imaging, follow-up visits, or treatment scheduling may have contributed to disease progression in a subset of these patients. However, given the relatively small number of newly diagnosed cases and the absence of a pre-pandemic comparison group, this interpretation should be considered exploratory.

In the whole sample, overall MRI findings differed between the COVID and post-COVID periods. Tumor size distribution showed a higher proportion of medium-to-large tumors (T3–T4) during the COVID waves, whereas early-stage tumors (T1) were more frequently observed in the post-COVID period. Similarly, overall tumor stage distribution differed significantly between the COVID and post-COVID periods. Because this analysis included both newly diagnosed and follow-up patients, these findings should be interpreted as reflecting overall MRI findings across the study periods rather than changes in stage at initial diagnosis alone. These overall imaging patterns may reflect changes in healthcare utilization during the pandemic, although this interpretation should be considered cautiously because the analysis included both newly diagnosed and follow-up patients. This pattern may also reflect the recovery of diagnostic activity after the pandemic. During the early COVID-19 waves, MRI examinations more frequently demonstrated larger tumors, whereas the post-COVID period showed a higher proportion of T1-stage tumors. While we cannot establish a cause-and-effect relationship, these findings are consistent with the gradual resumption of diagnostic pathways following pandemic-related disruptions in healthcare.

Given the relatively small number of newly diagnosed cases, these findings should be interpreted as descriptive trends rather than definitive evidence of causal effects.

The rate of documented missed treatments remained low, affecting 3.8% of patients during the COVID period and 3.8% in the post-COVID period, with no statistically significant differences between waves. This may partly explain why tumors in follow-up patients were more likely to show regression (66 patients) than progression (31 patients). Fortunately, relatively few treatments were missed due to the pandemic in our cohort. In contrast, other studies have reported substantial delays and disruptions in oncologic care during the COVID-19 period, which were associated with disease progression (20). This may reflect the fact that oncologic care in Hungary remained relatively accessible despite the pandemic, compared with international reports.

An additional observation was that Stage IV/C disease in the follow-up group was noted only in female patients. Although the number of cases was small and no statistical comparison was made, this finding may require further investigation in larger multicenter studies to see if there are sex-related differences in advanced colorectal cancer progression after healthcare disruptions.

## 5 Limitation

The major limitation is the relatively small sample size of the newly diagnosed patients which may limit statistical power, particularly after stratification by tumor stage. One of the most significant limitations of our research was that we lacked data from the first wave of the COVID-19 epidemic. This was because the MRI unit at Szent Margit Hospital only began operating during the pandemic, in September 2020. This period would have been particularly important for examining the effects on colorectal cancer diagnostics, as the decrease in the number of newly detected tumors was most likely to have occurred in the initial phase of the epidemic, in the first wave. At that time, the novelty of the epidemic and the associated uncertainty caused substantial fear within the population, which in many cases led to the avoidance of hospital care. The avoidance of healthcare facilities was especially reflected in the lack of diagnostic examinations and the reduced number of newly diagnosed colorectal cancer cases – an effect which we could have analyzed in greater detail had data from this period been available. Importantly, the absence of pre-pandemic baseline data limits causal interpretation, as the observed changes cannot be conclusively attributed to the COVID-19 pandemic and may also reflect natural temporal variation or institutional changes.

An additional limitation of this study is that tumor staging was based on radiological assessment (cTNM) rather than pathological staging (pTNM). Radiological staging may not fully correspond to surgical pathology, particularly with respect to nodal involvement. However, this limitation is partly attributable to the COVID-19 pandemic, during which surgical procedures were frequently delayed or postponed. As the primary focus of the present study was on tumor characteristics at the time of diagnostic imaging, radiological staging was considered the most appropriate and consistently available method for evaluating disease stage.

Furthermore, the accuracy of the study and the reliability of the results could have been significantly increased by the inclusion of a pre-pandemic control group. Establishing such a control group would have enabled comparisons between the pre-COVID-19 period and the pandemic period, providing a more precise assessment of how the pandemic influenced colorectal cancer diagnostics, treatment pathways, and morbidity.

In addition, the site of the main lesion (either colon or rectum) was not consistently documented in the study database. Therefore, no sub-analysis by tumor site was possible.

## 6 Conclusions

Overall, our results suggest that the COVID-19 pandemic period was associated with changes in colorectal cancer diagnostic activity, including a lower number of newly diagnosed cases and differences in tumor characteristics observed during this period. Following the pandemic, diagnostic activity recovered, and early-stage tumor detection increased compared with the COVID period. These findings underscore the importance of maintaining access to essential oncologic diagnostics and continuity of cancer care during public health emergencies. Even under restrictive conditions, prioritizing symptomatic patients, preserving follow-up imaging for high-risk cases, and ensuring sufficient diagnostic capacity may help mitigate diagnostic delays and prevent stage migration in future pandemic-like situations. These findings should be interpreted with caution, as the absence of pre-pandemic baseline data limits causal inference.

## Data Availability

The original contributions presented in the study are included in the article/**Supplementary Material**. Further inquiries can be directed to the corresponding author.
