# Supplementary material for: Impact of COVID-19 pandemic waves on colorectal cancer stage migration and diagnostic delays
Source: Front Oncol. 2026 Jul 29;16:1837204. doi: 10.3389/fonc.2026.1837204 (PMC13461719; doi:10.3389/fonc.2026.1837204)
Supplement: Supplementary file 1 [file Table1.docx]

**Supplementary Table S1.** Basics of TNM classification in colorectal cancer, adapted from AJCC 8th edition

| **Tumor** |  |  |
| --- | --- | --- |
| T1 | The cancer has grown through the muscularis mucosa into the submucosa. |  |
| T2 | The cancer has grown into the muscularis propria. |  |
| T3 | The cancer has grown into the outermost layers of the colon or rectum but has not gone through them. |  |
| T4 |  |  |
| T4a | The cancer has grown through the wall of the colon or rectum but has not grown into other nearby tissues or organs. |  |
| T4b | The cancer has grown through the wall of the colon or rectum and is attached to or has grown into other nearby tissues or organs. |  |
| **Nodes** |  |  |
| N0 | It has not spread to nearby lymph nodes. |  |
| N1 |  |  |
| N1a/N1b | It has spread to 1 to 3 nearby lymph nodes. |  |
| N1c | It has spread into areas of fat near the lymph nodes but not the nodes themselves. |  |
| N2 |  |  |
| N2a | It has spread to 4 to 6 nearby lymph nodes. |  |
| N2b | It has spread to 7 or more nearby lymph nodes. |  |
| **Metastasis** |  |  |
| M0 | It has not spread to distant sites. |  |
| M1 |  |  |
| M1a | It has spread to 1 distant organ (such as the liver or lung) or distant set of lymph nodes, but not to distant parts of the peritoneum (the lining of the abdominal cavity). |  |
| M1b | It has spread to more than 1 distant organ (such as the liver or lung) or distant set of lymph nodes, but not to distant parts of the peritoneum (the lining of the abdominal cavity). |  |
| M1c | It has spread to distant parts of the peritoneum (the lining of the abdominal cavity), and may or may not have spread to distant organs or lymph nodes. |  |

**Supplementary Table S2.** Descriptive characteristics of the patients in the whole sample (n=198).

| **Variable** | **Total** | | **New cases** | | **Follow-up** | |
| --- | --- | --- | --- | --- | --- | --- |
|  | **New cases** | **Follow-up** | **Male** | **Female** | **Male** | **Female** |
| Number of subjects (males) | 43 (20) | 155 (70) | 20 | 23 | 70 | 85 |
| Age mean (±SD), years | 68.98 (±11.8) | 64.97 (±12.23) | 68.65 (±13.75) | 69.26 (±10.1) | 61.09 (±10.3) | 68.16 (±10.3) |
| Time between the most recent previous examination and the COVID-period scan (±SD), months | - | 6.1 (±8) | - | - | 8.54 (±6.7) | 9.29 (±8.95) |
| Stage in previous scan, n (%) |  |  | - | - |  |  |
| I. | - | 56 (36.1%) | - | - | 22 (31.4%) | 34 (40%) |
| II. | - | 28 (18.1%) | - | - | 12 (17.1%) | 16 (18.8%) |
| II/A | - | 18 (11.6%) | - | - | 7 (10%) | 11 (13%) |
| II/B | - | 9 (5.8%) | - | - | 4 (5.7%) | 5 (5.9%) |
| II/C | - | 1 (0.6%) | - | - | 1 (1.4%) | 0 |
| III. | - | 37 (23.9%) | - | - | 19 (27.1%) | 18 (21.2%) |
| III/A | - | 11 (7.1%) | - | - | 4 (5.7%) | 7 (8.2%) |
| III/B | - | 22 (14.2%) | - | - | 13 (18.6%) | 9 (10.6%) |
| III/C | - | 4 (2.6%) | - | - | 2 (2.9%) | 2 (2.4%) |
| IV. | - | 34 (21.9%) | - | - | 17 (24.3%) | 17 (20%) |
| IV/A | - | 20 (12.9%) | - | - | 14 (20%) | 6 (7.1%) |
| IV/B | - | 3 (1.9%) | - | - | 2 (2.8%) | 1 (1.2%) |
| IV./C | - | 11 (7.1%) |  |  | 1 (1.4%) | 10 (11.8%) |
| Tumor in previous scan, n (%) |  |  |  |  |  |  |
| T1 | - | 59 (38.1%) | - | - | 23 (32.9%) | 36 (42.4%) |
| T2 | - | 24 (15.5%) | - | - | 13 (18.6%) | 11 (12.9%) |
| T3 | - | 52 (33.5%) | - | - | 26 (37.1%) | 26 (30.6%) |
| T4 | - | 20 (12.9%) | - | - | 8 (11.4%) | 12 (14.1%) |
| T4a | - | 16 (10.3%) | - | - | 7 (10%) | 9 (10.6%) |
| T4b | - | 4 (2.6%) | - | - | 1 (1.4%) | 3 (3.5%) |
| Nodes in previous scan, n (%) |  |  |  |  |  |  |
| N0 | - | 94 (60.6%) | - | - | 41 (58.6%) | 53 (62.4%) |
| N1 | - | 40 (25.8%) | - | - | 17 (24.3%) | 23 (27.1%) |
| N1a | - | 16 (10.3%) | - | - | 8 (9.4%) | 8 (11.4%) |
| N1b | - | 15 (9.7%) | - | - | 6 (8.6%) | 9 (10.6%) |
| N1c | - | 9 (5.8%) | - | - | 3 (4.3%) | 6 (7.1%) |
| N2 | - | 21 (13.5%) | - | - | 12 (17.1%) | 9 (10.6%) |
| N2a | - | 11 (7.1%) | - | - | 5 (7.1%) | 6 (7.1%) |
| N2b | - | 10 (6.5%) | - | - | 7 (10%) | 3 (3.5%) |
| Metastasis in previous scan, n (%) |  |  |  |  |  |  |
| M0 | - | 120 (77.4%) | - | - | 52 (74.3%) | 68 (80%) |
| M1 | - | 35 (22.6%) | - | - | 18 (25.7%) | 17 (20%) |
| M1a | - | 21 (13.5%) | - | - | 14 (20%) | 7 (8.2%) |
| M1b | - | 4 (2.6%) | - | - | 3 (4.3%) | 1 (1.2%) |
| M1c | - | 10 (6.5%) | - | - | 1 (1.4%) | 9 (10.6%) |
| Stage in recent scan, n (%) |  |  |  |  |  |  |
| I. | 19 (44.2%) | 89 (57.4%) | 9 (45%) | 10 (43.5%) | 37 (52.9%) | 52 (61.2%) |
| II. | 5 (11.6%) | 12 (7.7%) | 2 (10%) | 3 (13%) | 4 (5.7%) | 8 (9.4%) |
| II/A | 4 (9.3%) | 8 (5.2%) | 1 (5%) | 3 (13%) | 3 (4.3%) | 5 (5.9%) |
| II/B | 0 | 4 (2.6%) | 0 | 0 | 1 (1.4%) | 3 (3.5%) |
| II/C | 1 (2.3%) | 0 | 1 (5%) | 0 | 0 | 0 |
| III. | 19 (44.2%) | 10 (6.4%) | 9 (45%) | 10 (43.5%) | 6 (8.6%) | 4 (4.7%) |
| III/A | 8 (18.6%) | 7 (4.5%) | 3 (15%) | 5 (21.7%) | 4 (5.7%) | 3 (3.5%) |
| III/B | 6 (14%) | 3 (1.9%) | 5 (25%) | 1 (4.3%) | 2 (2.9%) | 1 (1.2%) |
| III/C | 5 (11.6%) | 0 | 1 (5%) | 4 (17.4%) | 0 | 0 |
| IV. | 0 | 44 (28.4%) | 0 | 0 | 23 (32.9%) | 21 (24.7%) |
| IV/A | 0 | 27 (17.4%) | 0 | 0 | 19 (27.1%) | 8 (9.4%) |
| IV/B | 0 | 6 (3.9%) | 0 | 0 | 4 (5.7%) | 2 (2.4%) |
| IV./C | 0 | 11 (7.1%) | 0 | 0 | 0 | 11 (12.9%) |
| Tumor in recent scan, n (%) |  |  |  |  |  |  |
| T1 | 17 (39.5%) | 107 (69%) | 6 (30%) | 11 (47.8%) | 47 (67.1%) | 60 (70.6%) |
| T2 | 11 (25.6%) | 13 (8.4%) | 7 (35%) | 4 (17.4%) | 9 (12.9%) | 4 (4.7%) |
| T3 | 9 (20.9%) | 19 (12.3%) | 5 (25%) | 4 (17.4%) | 7 (10%) | 12 (14.1%) |
| T4 | 6 (14%) | 16 (10.3%) | 2 (10%) | 4 (17.4%) | 7 (10%) | 9 (10.6%) |
| T4a | 1 (2.3%) | 15 (9.7%) | 0 | 1 (4.3%) | 7 (10%) | 8 (9.4%) |
| T4b | 5 (11.6%) | 1 (0.6%) | 2 (10%) | 3 (13%) | 0 | 1 (1.2%) |
| Nodes in recent scan, n (%) |  |  |  |  |  |  |
| N0 | 24 (55.8%) | 117 (75.5%) | 11 (55%) | 13 (56.5%) | 53 (75.7%) | 64 (75.3%) |
| N1 | 10 (23.3%) | 27 (17.4%) | 4 (20%) | 6 (26%) | 10 (14.3%) | 17 (20%) |
| N1a | 3 (7%) | 11 (7.1%) | 1 (5%) | 2 (8.7%) | 5 (7.1%) | 6 (7.1%) |
| N1b | 7 (16.3%) | 13 (8.4%) | 3 (15%) | 4 (17.4%) | 5 (7.1%) | 8 (9.4%) |
| N1c | 0 | 3 (1.9%) | 0 | 0 | 0 | 3 (3.5%) |
| N2 | 9 (20.9%) | 11 (7.1%) | 5 (25%) | 4 (17.4%) | 7 (10%) | 4 (4.7%) |
| N2a | 6 (14%) | 4 (2.6%) | 4 (20%) | 2 (8.7%) | 2 (2.9%) | 2 (2.4%) |
| N2b | 3 (7%) | 7 (4.5%) | 1 (5%) | 2 (8.7%) | 5 (7.1%) | 2 (2.4%) |
| Metastasis in previous scan, n (%) |  |  |  |  |  |  |
| M0 | 43 (100%) | 110 (71%) | 20 (100%) | 23 (100%) | 47 (67.1%) | 63 (74.1%) |
| M1 | 0 | 45 (29%) | 0 | 0 | 23 (32.9%) | 22 (25.9%) |
| M1a | 0 | 28 (18.1%) | 0 | 0 | 19 (27.1%) | 9 (10.6%) |
| M1b | 0 | 6 (3.9%) | 0 | 0 | 4 (5.7%) | 2 (2.4%) |
| M1c | 0 | 11 (7.1%) | 0 | 0 | 0 | 11 (12.9%) |
| Tumor response, n (%) |  |  |  |  |  |  |
| Progression | - | 31 (20%) | - | - | 14 (20%) | 17 (20%) |
| Regression | - | 66 (42.6%) | - | - | 30 (42.9%) | 36 (42.4%) |
| Stable disease | - | 58 (37.4%) | - | - | 26 (37.1%) | 32 (37.6%) |
| Missed Treatment, n (%) | - | 9 (5.8%) | - | - | 2 (2.9%) | 7 (8.2%) |
| Recurrence, n (%) | - | 8 (5.2%) | - | - | 4 (5.7%) | 4 (4.7%) |

**Supplementary Table S3.** Wave-specific stage distribution of newly diagnosed colorectal cancer cases across the different COVID-19 waves (n = 43).

| **Stage** | **COVID first waves, n** | | **COVID late waves + post-COVID period, n** | | | **Total** |
| --- | --- | --- | --- | --- | --- | --- |
|  | **Wave 2** | **Wave 3** | **Wave 4** | **Wave 5** | **Post-COVID** |  |
| Stage I. | 2 | 1 | 3 | 0 | 13 | 19 |
| Stage II. | 1 | 1 | 0 | 1 | 2 | 5 |
| II./A | 1 | 1 | 0 | 1 | 1 | 4 |
| II./B | 0 | 0 | 0 | 0 | 0 |  |
| II./C | 0 | 0 | 0 | 0 | 1 | 1 |
| Stage III. | 2 | 3 | 4 | 0 | 10 | 19 |
| III./A | 1 | 0 | 2 | 0 | 5 | 8 |
| III./B | 0 | 2 | 2 | 0 | 2 | 6 |
| III./C | 1 | 1 | 0 | 0 | 3 | 5 |
| Stage IV. | 0 | 0 | 0 | 0 | 0 | 0 |
| IV./A | 0 | 0 | 0 | 0 | 0 | 0 |
| IV./B | 0 | 0 | 0 | 0 | 0 | 0 |
| IV./C | 0 | 0 | 0 | 0 | 0 | 0 |

**Supplementary Table S4.** Wave-specific tumor size distribution during and after the COVID-19 period in the whole sample (n = 198).

| **Tumor size** | **COVID first waves, n** | | **COVID late waves + post-COVID period, n** | | | **Total** |
| --- | --- | --- | --- | --- | --- | --- |
|  | **Wave 2** | **Wave 3** | **Wave 4** | **Wave 5** | **Post-COVID** |  |
| T1 | 11 | 11 | 14 | 10 | 78 | 124 |
| T2 | 4 | 4 | 3 | 1 | 12 | 24 |
| T3 | 8 | 5 | 9 | 1 | 5 | 28 |
| T4 | 6 | 7 | 0 | 0 | 9 | 22 |
| T4a | 4 | 6 | 0 | 0 | 6 | 16 |
| T4b | 2 | 1 | 0 | 0 | 3 | 6 |
